# Extracellular Vesicles in Luminal Fluid of the Ovine Uterus

**DOI:** 10.1371/journal.pone.0090913

**Published:** 2014-03-10

**Authors:** Gregory Burns, Kelsey Brooks, Mark Wildung, Raphatphorn Navakanitworakul, Lane K. Christenson, Thomas E. Spencer

**Affiliations:** 1 Department of Animal Sciences, Washington State University, Pullman, Washington, United States of America; 2 School of Molecular Biosciences, Washington State University, Pullman, Washington, United States of America; 3 Department of Molecular and Integrative Physiology, University of Kansas Medical School, Kansas City, Kansas, United States of America; The University of Georgia, United States of America

## Abstract

Microvesicles and exosomes are nanoparticles released from cells and can contain small RNAs, mRNA and proteins that affect cells at distant sites. In sheep, endogenous beta retroviruses (enJSRVs) are expressed in the endometrial epithelia of the uterus and can be transferred to the conceptus trophectoderm. One potential mechanism of enJSRVs transfer from the uterus to the conceptus is via exosomes/microvesicles. Therefore, studies were conducted to evaluate exosomes in the uterine luminal fluid (ULF) of sheep. Exosomes/microvesicles (hereafter referred to as extracellular vesicles) were isolated from the ULF of day 14 cyclic and pregnant ewes using ExoQuick-TC. Transmission electron microscopy and nanoparticle tracking analysis found the isolates contained vesicles that ranged from 50 to 200 nm in diameter. The isolated extracellular vesicles were positive for two common markers of exosomes (CD63 and HSP70) by Western blot analysis. Proteins in the extracellular vesicles were determined by mass spectrometry and Western blot analysis. Extracellular vesicle RNA was analyzed for small RNAs by sequencing and enJSRVs RNA by RT-PCR. The ULF extracellular vesicles contained a large number of small RNAs and miRNAs including 81 conserved mature miRNAs. Cyclic and pregnant ULF extracellular vesicles contained enJSRVs *env* and *gag* RNAs that could be delivered to heterologous cells *in vitro*. These studies support the hypothesis that ULF extracellular vesicles can deliver enJSRVs RNA to the conceptus, which is important as enJSRVs regulate conceptus trophectoderm development. Importantly, these studies support the idea that extracellular vesicles containing select miRNAs, RNAs and proteins are present in the ULF and likely have a biological role in conceptus-endometrial interactions important for the establishment and maintenance of pregnancy.

## Introduction

Establishment of pregnancy in domestic ruminants (i.e., sheep, cattle, goats) begins at the conceptus stage and includes pregnancy recognition signaling, implantation, and placentation [1]–[4]. In sheep, the morula-stage embryo enters the uterus on day 4 to 6 post-mating and continues development to a blastocyst. After hatching from the zona pellucida (day 8), the blastocyst slowly grows into a tubular or ovoid form referred to as a conceptus (i.e., embryo/fetus and associated extraembryonic membranes). Elongation of the ovoid (∼5–10 mm) conceptus begins on day 12. By day 16, the filamentous conceptus (10 to 15 cm) occupies the entire length of the uterine horn ipsilateral to the corpus luteum, thus preventing luteolysis and signaling maternal recognition of pregnancy. Although blastocysts can develop entirely *in vitro*, they must be transferred into a receptive uterus for growth and development into an elongated conceptus [5]. The endometrium of the uterus secretes substances, collectively termed histotroph, that govern growth and elongation of the conceptus via effects on trophectoderm proliferation and migration as well as attachment and adhesion to the endometrial luminal epithelium (LE) [1], [6], [7]. Histotroph is derived primarily from transport and/or synthesis and secretion of substances by the endometrial LE and glandular epithelia (GE) and is a complex and rather undefined mixture of proteins, lipids, amino acids, sugars and ions [8], [9]. The recurrent early pregnancy loss observed in uterine gland knockout (UGKO) ewes established the importance of uterine epithelial-derived histotroph for support of conceptus elongation and implantation [10]. Emerging evidence indicates that the endometrium and trophoblast of the placenta also release exosomes/microvesicles that are critical components of uterine luminal fluid (ULF) involved in conceptus-endometrial interactions during early pregnancy [11], [12].

Exosomes and microvesicles provide an alternative mode of cell-cell communication [13],[14]. They are preformed, membrane-covered nanovesicles (30–150 nm) of endocytic origin secreted by most cell types *in vitro*, including extravillous and villous human trophoblast cells, primary trophoblast from term human placenta, and ovine endometrial GE cells [11],[12],[15]–[19]. Extracellular vesicles have also been identified in many different bodily fluids including amniotic fluid, urine, and blood [13] as well as human ULF [11]. Exosomes have surface receptors/ligands of the original cells and thus the potential to selectively interact with specific target cells [20]. They contain numerous lipids and proteins as well as mRNAs and miRNAs [14], [21], [22]. Indeed, exosomes can horizontally transfer mRNAs to other cells, which can then be translated into functional proteins in the new location [14], [22], [23]. Similarly, miRNAs can be transferred via exosomes and regulate gene expression in recipient cells [22], [24]–[26]. For instance, microvesicles can elicit biological effects, such as increased cell migration [27], that is important for conceptus elongation in ruminants.

Endogenous retroviruses (ERVs) are present in the genome of all vertebrates and are transmitted in a Mendelian fashion [28]. Domestic sheep harbor at least 27 copies of ERVs in their genome, termed enJSRVs, because they are highly related to the exogenous and pathogenic Jaagsiekte sheep retrovirus (JSRV) [29], [30]. Many of the enJSRVs have an intact reading frame for the full-length envelope (*env*) gene, and several are completely intact for all genes and produce viral particles. The enJSRVs Env is essential for conceptus elongation in sheep, as conceptuses lacking enJSRVs Env did not elongate due to defective trophectoderm cell proliferation [31]. Of relevance to this study, we recently found that enJSRVs are transmitted from the sheep uterus to the conceptus trophectoderm using a trans-species embryo transfer model [32]. In that study, the transmission of enJSRVs was hypothesized to occur via the endometrium shedding enJSRV virus particles that entered the conceptus via cell surface receptors. Alternatively, enJSRVs could be delivered from the endometrium to conceptus trophectoderm via extracellular vesicles. Therefore, the present studies tested the hypothesis that extracellular vesicles are present in ULF and contain specific RNAs (enJSRVs RNA, mRNA, and miRNA) and proteins that may be utilized for communication between the endometrium and conceptus.

## Materials and Methods

### Animals

Mature Rambouillet female ewes (*Ovis aries*) were checked daily for the onset of estrus (designated as day 0) using a vasectomized ram. Ewes assigned to collection for pregnancy tissues were bred using an intact ram of proven fertility at estrus (day 0 = mating). The Institutional Animal Care and Use Committee of Washington State University approved all experimental and surgical procedures.

### Extracellular Vesicle Isolation

Uterine luminal fluid (ULF) was obtained from day 14 cyclic (C) or pregnant (P) ewes (n = 4–8 per status). Briefly, uteri were removed at necropsy, and the lumen was gently flushed with 20 ml sterile PBS (pH 7.2). The ULF was clarified by centrifugation at 21,000× *g* for 15 minutes and filtered through a 0.2 µm nylon filter. Extracellular vesicles were isolated from ULF by adding 200 µl ExoQuick-TC (System Biosciences, Cupertino, CA) precipitation solution to one ml of filtered ULF. The ULF with ExoQuick-TC was incubated overnight at 4°C and then centrifuged (1,500× *g* at 4°C for 30 min) to pellet the extracellular vesicles. The pellets were suspended in PBS or mammalian protein extraction reagent (M-PER, Thermo Scientific, Rockford, IL) containing HALT protease inhibitor cocktail (Thermo Scientific).

### Transmission Electron Microscopy

Extracellular vesicles, isolated from ULF and suspended in PBS (pH 7.2), were pipetted (5 µl) onto formvar-coated copper grids (FF200-Cu, Electron Microscopy Sciences, Hatfield, PA) and allowed to settle for 20 minutes at room temperature. Excess PBS was removed by wicking with filter paper before fixation using a 2% paraformaldehyde, 2% glutaraldehyde, and 0.05 M phosphate solution for 2 minutes. Grids were washed 3 times with distilled water prior to application of 1% phosphotungstic acid (PTA) counterstain for 1 minute. Excess liquid was removed by wicking with filter paper, and the grids were allowed to dry overnight at room temperature. Grids were analyzed using a Technai G2 20 transmission electron microscope (FEI, Hillsboro, OR).

### Nanoparticle Tracking Analysis

Nanoparticle tracking analysis of extracellular vesicles, isolated from ULF and suspended in PBS+0.1% BSA (pH 7.2), was performed using a NanoSight LM10-HS (NanoSight Ltd., Amesbury, UK) instrument calibrated with 50 nm polystyrene beads (Polysciences, Warrington, PA). Particle suspensions were diluted with PBS to attain a concentration of 1–8×10^8^ particles per milliliter for analysis. Videos were recorded for 60 seconds during which the nanoparticle tracking analysis software (NanoSight Ltd., Amesbury, UK) tracked each visible particle. The Stokes-Einstein equation was employed to determine the size distribution and number of particles (concentration) within the sample.

### Western Blot Analysis

Extracellular vesicle isolates were suspended in 40 µl of M-PER (Thermo Scientific) with HALT protease inhibitor cocktail (Thermo Scientific) for 15 minutes on a tube rotator at room temperature. Protein concentration was determined by A_280_ measurements using a NanoDrop 2000 (Thermo Scientific). Lysates were then mixed with Laemmli sample buffer (31.5 mM Tris-HCl, pH 6.8; 10% glycerol; 5% β-mercaptoethanol; 1% SDS; 0.01% bromophenol blue), denatured at 95°C for 5 minutes, and separated by SDS-PAGE at a constant voltage of 150 V for approximately 90 minutes in 1× running buffer (25 mM Tris, 192 mM glycine, 0.1% SDS). Proteins were transferred to 0.45 µm Protran BA 85 nitrocellulose membrane (GE Healthcare, Buckinghamshire, UK) in Towbin transfer buffer (25 mM Tris, 192 mM glycine, 20% methanol) at 100 V for 60 minutes. Membranes were placed in blocking buffer (TBS, 5% non-fat milk, 0.1% Tween 20) for 1 hour at room temperature. Primary antibodies [CD63 (1∶1000, Cat # EXOAB-CD63A-1, System Biosciences), HSP70 (1∶1000, Cat # EXOAB-HSP70A-1, System Biosciences), CTSL1 (1∶300, Cat # 3912-100, BioVision, Mountain View, CA), or PTGS2 (1∶200, Cat # LS-C150711, LifeSpan Biosciences, Inc., Seattle, WA)] were diluted in blocking buffer and incubated with the blots overnight at 4°C. Membranes were washed with TBS containing 0.1% Tween 20 (TBST) before incubation with goat anti-rabbit HRP conjugated secondary antibody (System Biosciences) at 1∶10,000 dilution for 1 hour at room temperature. Membranes were washed with excess TBST and incubated with SuperSignal West Pico Chemiluminescent Substrate (Thermo Scientific) for 3 minutes prior to imaging with a ChemiDoc MP system and Image Lab 4.1 software (BioRad, Hercules, CA).

### Mass Spectrometry Analysis

The protein concentration of extracellular vesicles, isolated from ULF and suspended in PBS (pH 7.2), was determined using a QuickStart Bradford assay (BioRad). Mass spectrometry (MS) analysis was conducted by Applied Biomics (Hayward, CA, USA). Briefly, a pool of extracellular vesicle protein (15 µg total), from day 14 cyclic or pregnant ewes (n = 4 ewes per pool), was exchanged into 50 mM ammonium bicarbonate buffer, and DTT was then added to a final concentration of 10 mM. Samples were incubated at 60°C for 30 min followed by cooling to room temperature. Iodoacetamide was added to a final concentration of 10 mM and incubated in the dark for 30 min at room temperature. A tryptic digestion was performed at 37°C overnight. Nano liquid chromatography (LC) was carried out using a Dionex Ultimate 3000 system (Milford, MA, USA). Tryptic peptides were loaded into an α-Precolumn Cartridge and separated using an acetonitrile gradient (ranging from 5% to 60%) on the Nano LC column. Fractions were collected at 20 sec intervals followed by Mass Spectrometry analysis on AB SCIEX TOF/TOF 5800 System (AB SCIEX, Framingham, MA, USA). Mass spectra were acquired in reflectron positive ion mode. Time of flight (TOF)/TOF tandem MS fragmentation spectra were acquired for each ion, averaging 4000 laser shots per fragmentation spectrum (excluding trypsin autolytic peptides and other known background ions). Identification of the resulting peptide mass and the associated fragmentation spectra were submitted to GPS Explorer workstation equipped with MASCOT search engine (Matrix Science, London, UK) to search the non-redundant database of National Center for Biotechnology Information (NCBInr). Searches were performed without constraining protein molecular weight or isoelectric point, with variable carbamidomethylation of cysteine and oxidation of methionine residues, and with one missed cleavage allowed in the search parameters.

### RNA Isolation and Analysis of enJSRVs

Total RNA was isolated from frozen endometrium, conceptus or isolated ULF extracellular vesicles in PBS with Qiazol lysis reagent (Qiagen, Valencia, CA) and processed with the miRNeasy kit (Qiagen). DNase I (Qiagen) treatment was performed on-column according to manufacturer's instructions, and samples were stored at −80°C in nuclease-free water. The RNA concentration was determined by spectrophotometry analysis with a NanoDrop 2000 instrument (Thermo Scientific). The RNA profiles were collected using an Agilent Bioanalyzer 2100 (Agilent Technologies, Santa Clara, CA) with RNA 6000 Nano and Small RNA Labchip kits (Agilent Technologies) according to manufacturer's instructions.

Total RNA was reverse transcribed with using the iScript cDNA synthesis kit (BioRad) according to the manufacturer's instructions and stored at −20°C for further analysis. Genomic DNA contamination was tested by inclusion of mixtures without reverse transcriptase enzyme. The cDNA was used in subsequent PCR reactions with TaKaRa hot start DNA polymerase (Clontech Laboratories, Inc., Mountain View, CA) and primers designed to target enJSRVs *env* (forward: GRY TTT CCR TGG GAT AAG GTG AA, reverse: ACA ATC ACY AGA CCC TTA CCA TTG) and *gag* (forward: GTG CTG ACA TTG GAC CCT, reverse: CAC AAA CAA AGC AAC CCG AA) RNA under the following conditions: 95°C for 2 minutes; 35 cycles of 98°C for 10 seconds, 55°C for 30 seconds, 72°C for 1 minute; and a final step of 72°C for 2 minutes. The PCR products were separated in a 1% agarose in TAE gel containing ethidium bromide and visualized using a ChemiDoc MP system and Image Lab 4.1 software (BioRad).

### Small RNA Sequencing and Analysis

The RNA, extracted from extracellular vesicles isolated from ULF of cyclic (n = 4) and pregnant (n = 4) ewes, was pooled for a total of 5–10 ng small RNA with less than 1 µg of total RNA per sample. Small RNA sequencing was performed by the Washington State University Genomics Core using the Ion Torrent platform (Life Technologies). Hybridization, RNA ligation and reverse transcription were performed using Ion Total RNA-Seq Kit v2 (Cat # 4475936, Life Technologies) according to the manufacturer's instructions for small RNA sequencing. Purification and size selection of the cDNA was conducted using AMPureXP beads (Beckmann-Coulter Genomics, Brea, CA). Libraries were quantified by real-time quantitative PCR (qPCR) and pooled into a single emulsion PCR reaction performed using an Ion One Touch instrument (Life Technologies). Sequencing bead numbers, quality, and enrichment for DNA carrying beads was assessed by flow cytometry using a Millipore Guava (EMD Millipore Corporation, Billerica, MA). Beads were then sequenced using a 316D semi-conductor sequencing chip.

Sequences were analyzed with CLC Genomics Workbench software (CLC Bio, Aarhus, Denmark) using the small RNA sequencing workflow restricting sequences to 15–55 bp, less than 2 mismatches, 10 or more sequence copies, referencing the miRBase (release 19) database for *Bos taurus* mature miRNAs, and grouping by mature miRNA. Targets were predicted using miRNA_Targets (http://mamsap.it.deakin.edu.au/mirna_targets/) for *Bos taurus* based on mature miRNA ID with the miRanda algorithm and an energy cutoff of −30. Gene ontology of the targeted genes was determined based on Ensembl IDs of the targets run using the Panther Classification System (http://www.pantherdb.org) selected for *Bos taurus*. The data discussed in this publication was deposited in NCBI's Gene Expression Omnibus (accession number GSE53974) [33].

### miRNA Analysis by qPCR

Total RNA from endometria, conceptuses, and ULF extracellular vesicles was isolated with the miRNeasy kit (Qiagen, Valencia, CA). The RNA was then polyadenylated, annealed to a universal sequence, and reverse transcribed with a miScript II RT kit (Qiagen) according to the manufacturer's instructions. Genomic DNA contamination was tested by inclusion of mixtures without reverse transcriptase enzyme. Quantitative real-time PCR reactions were assembled with 400 pg cDNA per reaction using the miScript SYBR Green PCR kit (Qiagen) with the BioRad CFX 96 Touch real time PCR machine (BioRad). Qiagen miScript mature miRNA primer assays (Cat # MS00031220, MS00003136, MS00004179, MS00031514, MS00031605, MS00003871, MS00009079, MS00031584, MS00003423) were used to amplify miRNA sequences. Reactions without template were used as negative controls to verify experimental results. The threshold line was set in the linear region of the amplification plot above the baseline noise and quantification cycle (Cq) values were determined as the cycle number in which the threshold line intersected the amplification curve. The miScript control primer assays SNORD95 and SNORD96A (Cat # MS00033726, MS00033733) were used as reference genes for data normalization. Expression values (2^−ΔΔCt^) were calculated using the geometric mean of SNORD95 and SNORD96A references and the geometric mean of the target gene Cq values.

### Vesicle Labeling and Delivery Analysis

Extracellular vesicles, collected from ULF with ExoQuick-TC as described, were labeled with a lipophilic green fluorescent dye (PKH67 from Sigma-Aldrich, St. Louis, MO). Extracellular vesicles suspended PBS were incubated with 1 ml of diluent mixed with PKH67 dye at final concentration of 5×10^−6^ M for 5 minutes. Fetal bovine serum (FBS) was added (2 ml) for 1 minute to stop the labeling reaction. The sample tube was filled with DMEM/F-12 media with 10% FBS, and extracellular vesicles were collected by ultracentrifugation for 30 minutes at 120,000× *g* at 4°C. Sample tubes containing pelleted extracellular vesicles were filled with DMEM/F-12 media containing 10% FBS and centrifuged two more times to remove excess dye. The final pellet was suspended in 100 µl DMEM/F-12 complete media and added directly to HEK 293T cells cultured in a 6-well plate or chamber slide. Prior to RNA collection or fixation for imaging, cells were washed vigorously two times with sterile PBS to remove any extraneous labeled vesicles.

Cells cultured for 48 hours in a 6-well plate were lysed with 1 ml Isol-RNA lysis reagent (5 PRIME, Inc., Gaithersburg, MD) for 5 minutes at room temperature. Chloroform (200 µl; Sigma-Aldrich) was added to the homogenate, mixed vigorously for 15 seconds and incubated for 3 minutes at room temperature. Samples were centrifuged at 12,000× *g* for 15 minutes at 4°C and the upper aqueous layer was removed. The RNA was precipitated with 500 µl 100% isopropanol, incubated at room temperature for 5 minutes and centrifuged at 12,000× *g* for 10 minutes at 4°C. The supernatant was removed, RNA washed with 1 ml 75% ethanol, and collected by centrifugation at 7,500× *g* for 5 minutes at 4°C. RNA was suspended in 20 µl RNase-free water and concentrations were measured with a NanoDrop 2000 instrument (Thermo Scientific). Reverse transcription of RNA and PCR analysis for the presence of enJSRVs were conducted as described above.

Chambers were removed from slides after 72 hours and cells were fixed with 4% paraformaldehyde at room temperature for 10 minutes before two, 5 minute washes in PBS. Two drops of VECTASHIELD HardSet mounting medium containing DAPI (Vector Laboratories, Inc., Burlingame, CA) was added for nuclear counterstaining and a glass coverslip was applied. Brightfield and epifluorescent (DAPI and FITC) images were collected using NIS Elements BR 3.2 software. The Z-stack images were collected with a Leica TCS SP8 X confocal microscope (Leica Microsystems, Mannheim, Germany) using system optimized stack thickness and sequential imaging of DAPI and FITC channels and Leica Application Suite Advanced Fluorescence (LAS AF) software. The 3D visualization module was used to render orthogonal sectioning, 3D images and video.

## Results

### Microvesicles and Exosomes are Present in ULF of the Ovine Uterus

**Figure 1:**
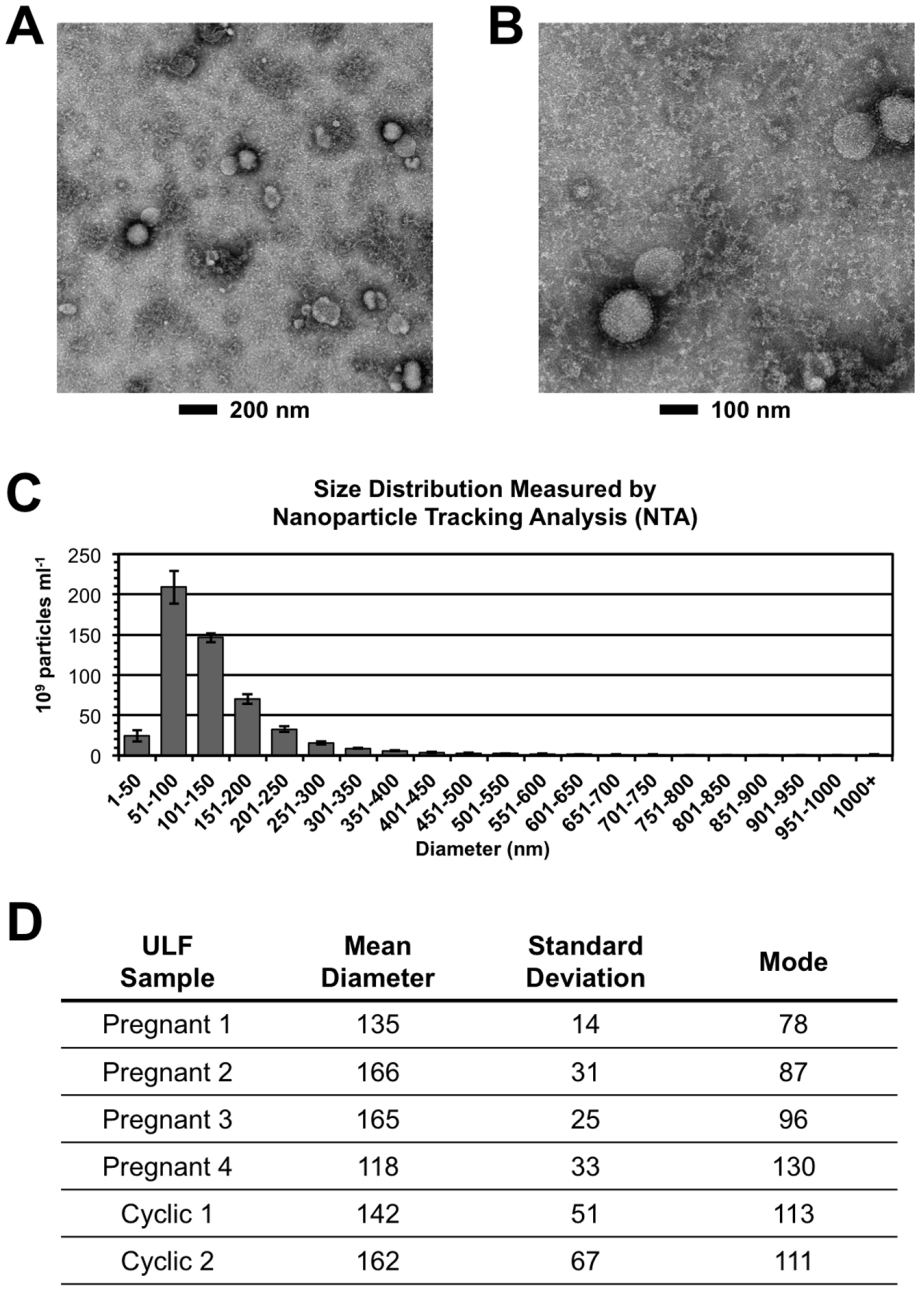
Transmission electron microscopy (TEM) and nanoparticle tracking analysis (NTA) of vesicles isolated from uterine luminal fluid of sheep. (A–B) TEM revealed vesicles of about 100 nm, consistent with exosomes. (C) NTA profile of extracellular vesicle collection from one individual. Measurements were collected from 3 samples; error bars represent +/−1 standard deviation. The profile confirms size estimates from TEM images. (D) Nanoparticle Tracking Analysis of collected extracellular vesicles. Precipitated extracellular vesicles were suspended in PBS+0.1%BSA and analyzed by NTA using a NanoSight instrument. Mean diameter, standard deviation of the mean, and mode size are reported (Replicates ≥3 per sample). These measurements are consistent with the presence of exosomes and microvesicles in the collected samples from ULF.

**Figure 2:**
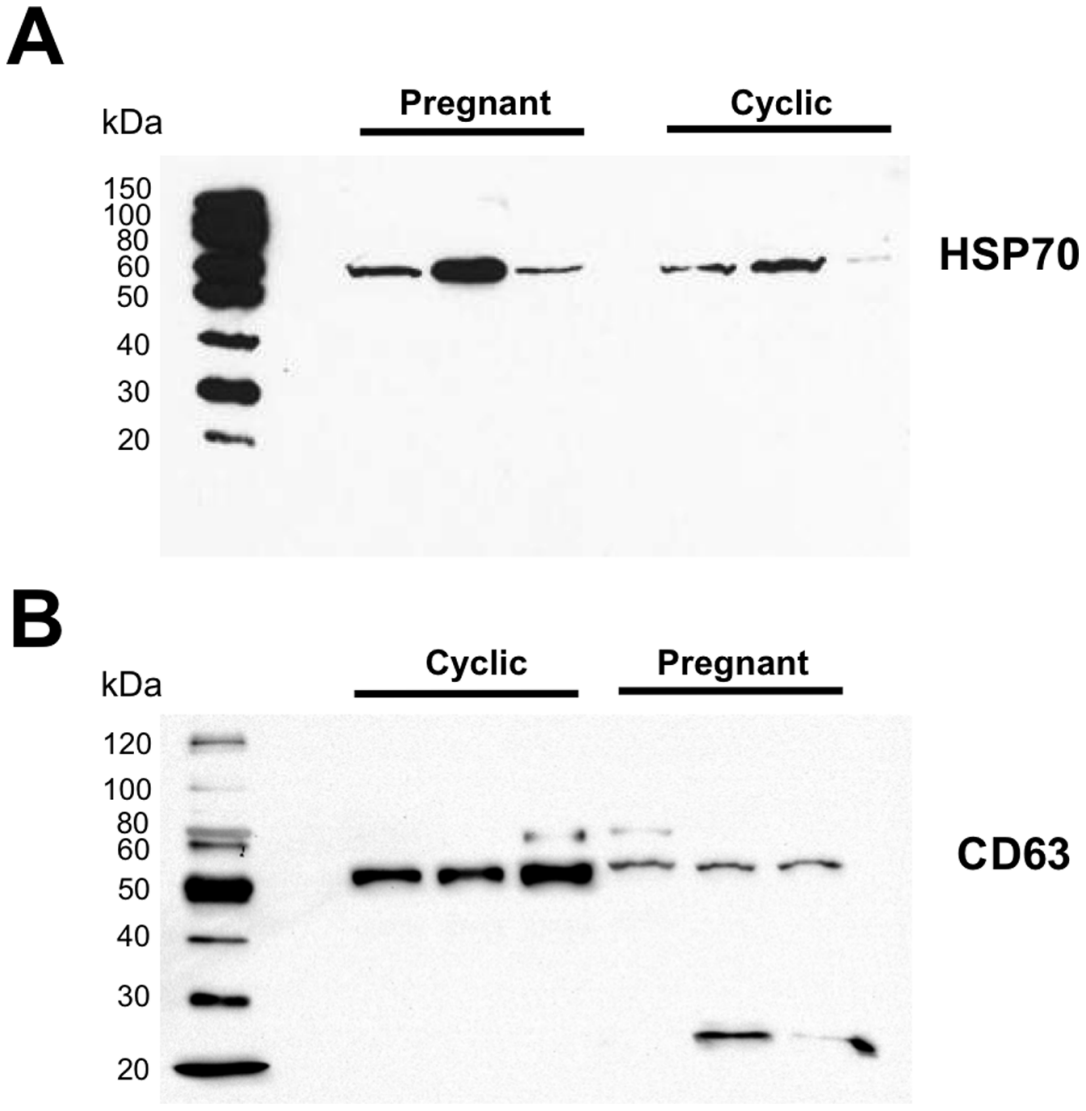
Western blot showing presence of HSP70 and CD63 in ULF derived extracellular vesicles from cyclic and pregnant ewes. Western blot analysis shows immunoreactive bands for (A) HSP70 and (B), CD63 two well characterized exosome markers, in all samples.

Extracellular vesicles were isolated from ULF of day 14 cyclic or pregnant ewes using ExoQuick-TC reagent. Transmission electron microscopy revealed the isolates contained vesicles approximately 100 nm in diameter (Fig. 1A and 1B). For accurate measurement of particle size and distribution, nanoparticle tracking analysis of the isolates was performed (Fig. 1C). This analysis revealed that the vesicles collected from ULF had an average diameter of 148 nm and an overall mode of about 100 nm (Fig. 1D). Western blot analysis revealed that the isolated vesicles from cyclic and pregnant ewes were positive for CD63 protein and heat shock protein 70 (HSP70) (Fig. 2), two well-characterized exosomal protein markers [34], [35].

### ULF Extracellular Vesicles Contain Proteins

**Figure 3:**
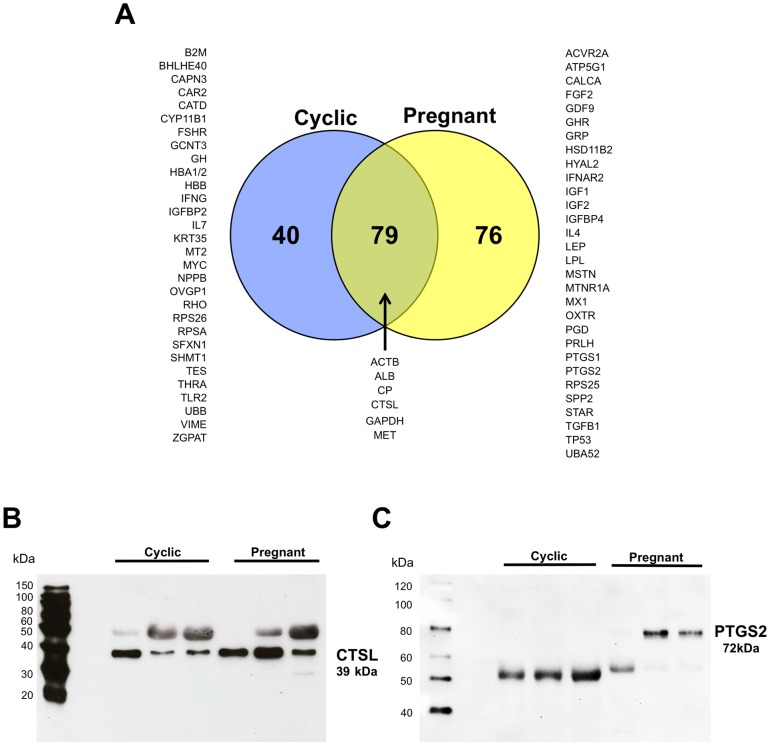
Proteomic analysis of cyclic and pregnant ULF extracellular vesicles. (A) Venn diagram containing 195 unique proteins identified by nano-LC-MS/MS analysis with 40 and 76 unique to the 14C and 14P groups respectively and 79 proteins in common. Note the presence of CTSL1 in both samples along with the presence of PTGS2 in only the 14P sample. (B) Western blot analysis shows the presence of CTSL in all samples. (C) PTGS2 immunoreactive bands are absent in 14C samples and present in 14P (2/3) samples.

Mass spectrometry (nano-LC-MS/MS) analysis of isolated ULF extracellular vesicles found many proteins expressed by the endometrial epithelia and/or conceptus trophectoderm including cathepsin L1 (CTSL1) and prostaglandin synthase two (PTGS2) (see Table S1). A total of 195 proteins were identified by nano-LC-MS/MS analysis with 40 and 76 unique to the cyclic and pregnant ewes, respectively (Fig. 3A). Comparison with the KEGG Brite exosome database and exosomal proteins reported in a variety of exosome studies resulted in five conserved proteins: GAPDH; TFAP2A, Transcription Factor AP-2 Alpha; RPS25, Ribosomal Protein S25; YWHAZ, Tyrosine 3-Monooxygenase/Tryptophan 5-Monooxygenase Activation Protein Zeta Polypeptide; ITGB1, Integrin beta 1 [13], [36], [37]. Western blot analysis confirmed the presence of CTSL1 in cyclic and pregnant extracellular vesicle samples (Fig. 3B). The antibody used for Western blot analysis is specific for PTGS2, which has a molecular weight of approximately 72 kDa, and has been documented to detect a non-specific immunoreactive band around 50 kDa. Indeed, PTGS2 protein was present in two of the three samples from pregnant ewes, and samples from cyclic ewes and one pregnant ewe displayed an immunoreactive protein of ∼52 kDa rather than the 72 kDa protein (Fig. 3C).

### ULF Extracellular Vesicles Contain Small RNAs and miRNAs

**Figure 4:**
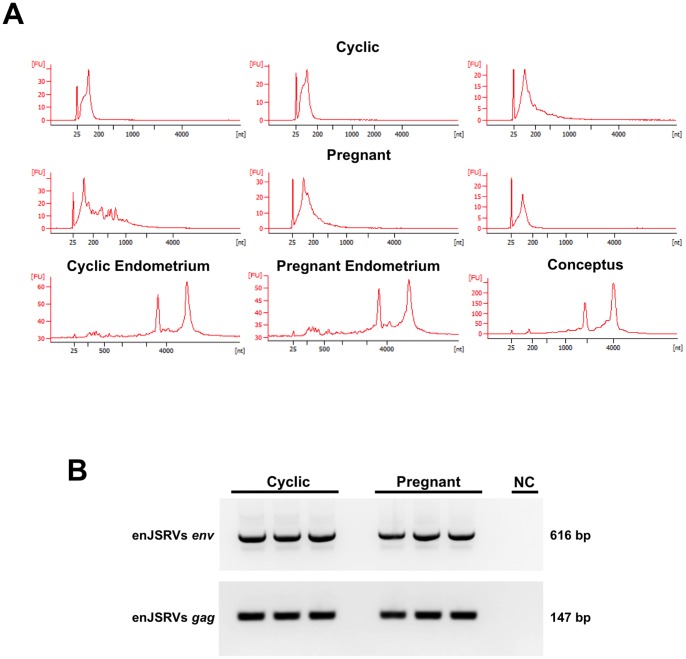
RNA content of extracellular vesicles. (A) The RNA profiles of extracellular vesicles, endometrium and conceptus samples using an Agilent 2100 Bioanalyzer. Note the cluster of RNA species at or below 200 base pairs in the extracellular vesicle samples both cyclic and pregnant; in contrast to endometrium and conceptus samples which display profiles characteristic of eukaryotic tissue with clearly defined 18S and 28S rRNA peaks and comparatively low percentages of transcripts below 200 base pairs. (B) Endogenous Jaagsiekte retrovirus (enJRSVs) *env* and *gag* RNA is present in extracellular vesicles from ULF of cyclic and pregnant ewes by RT-PCR. Day 14 conceptus and endometrium were used as positive controls for *env*. RT-PCR for *gag* utilized RNA from day 14 conceptus and 100 day placentome as positive controls. Negative control (NC) was performed by omitting cDNA in the reaction mix.

The RNA profiles of extracellular vesicles, endometrium and conceptus samples were collected using an Agilent 2100 Bioanalyzer (Fig. 4A). The analysis revealed that extracellular vesicles contained mostly small RNA species less than 200 bp in length, in contrast to the samples from endometrium and conceptus that contained larger RNA species including the 18S and 28S rRNAs. Next, small RNA sequencing was conducted using RNA isolated from ULF extracellular vesicles. Unannotated small RNA sequences accounted for greater than 80% of the reads in all three samples. These sequences are potential novel mature miRNAs involved in communication between the ovine conceptus trophectoderm and endometrial epithelia, in fact many of the unannotated sequences share homology with annotated miRNAs of other species by BLASTN searches of the mature miRNA miRBase.

**Figure 5:**
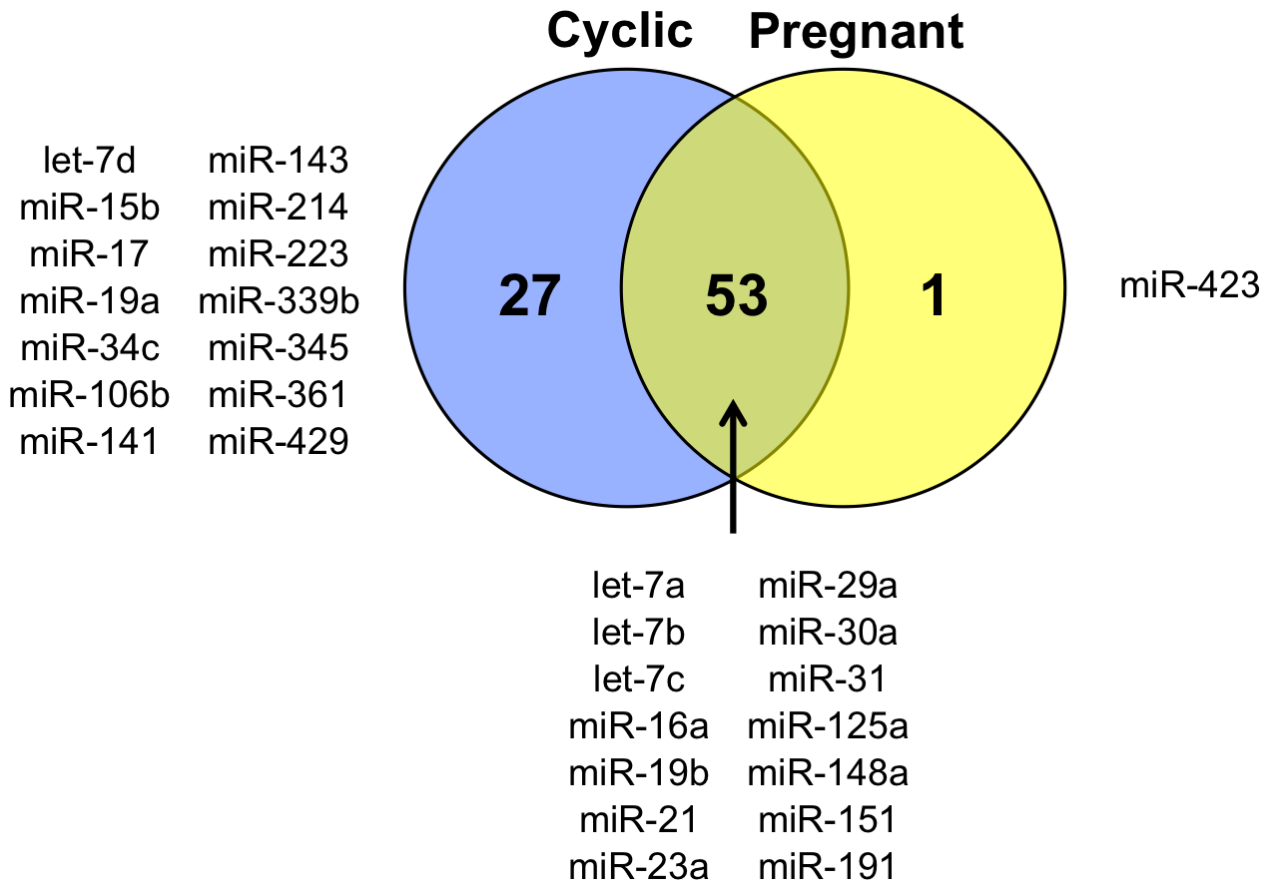
Small RNA sequencing of ULF derived extracellular vesicles. Next generation small RNA sequencing analysis restricted to at least 10 copies of each sequence from 15–55 nucleotides in length and grouped by mature miRNA produced 81 mature bovine miRNA species referenced in miRBase (Release 19). A total of 53 mature miRNAs were common to cyclic and pregnant samples with one unique to the 14P group and 27 unique to the 14C group.

**Figure 6:**
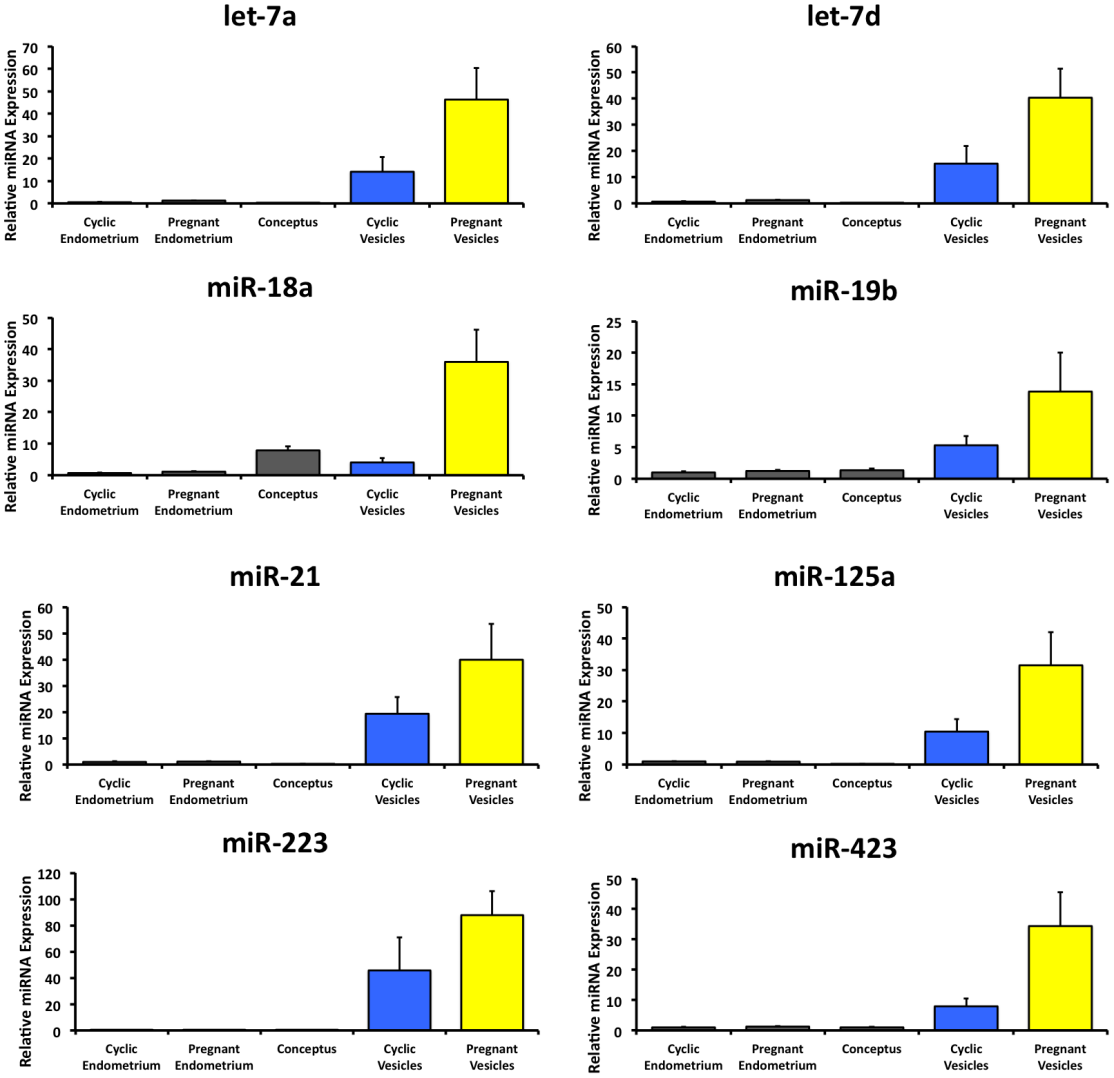
Cellular and extracellular detection of mature miRNA by RT-qPCR in cyclic and pregnant endometrium, conceptus and ULF extracellular vesicles. Presence of selected mature miRNAs was tested for in cyclic and pregnant endometrium, conceptus, and extracellular vesicles from cyclic and pregnant ewes using the Qiagen miScript system and mature miRNA primer assays (400 pg cDNA/well). Expression values (2^−ΔΔCt^) were calculated using the geometric mean of SNORD95 and SNORD96A references and the geometric mean of the target gene. Error bars represent ± 1 SEM.

Given the lack of sufficient transcriptomic and genomic data in sheep, the small RNAs were annotated using existing information from cattle. This analysis found 81 conserved mature miRNAs in the ULF extracellular vesicle small RNA sequences, with 53 in common between cyclic and pregnant samples and 1 unique annotated miRNA (bta-miR-423) in the day 14 pregnant samples (Fig. 5). The presence of 9 mature miRNA species (let-7a, led-7d, miR-18a, miR-19b, miR-21, miR-125a, miR-214, miR-223 and miR-423) was confirmed by RT-qPCR analysis of cyclic and pregnant ULF extracellular vesicles and compared to Day 14 endometrium and conceptus RNA (Fig. 6). Relative miRNA levels showed enrichment of miRNAs in conceptuses and ULF extracellular vesicles from pregnant and cyclic ewes.

### ULF Extracellular vesicles Contain enJSRVs RNA and Can Transmit Them to Cells

**Figure 7:**
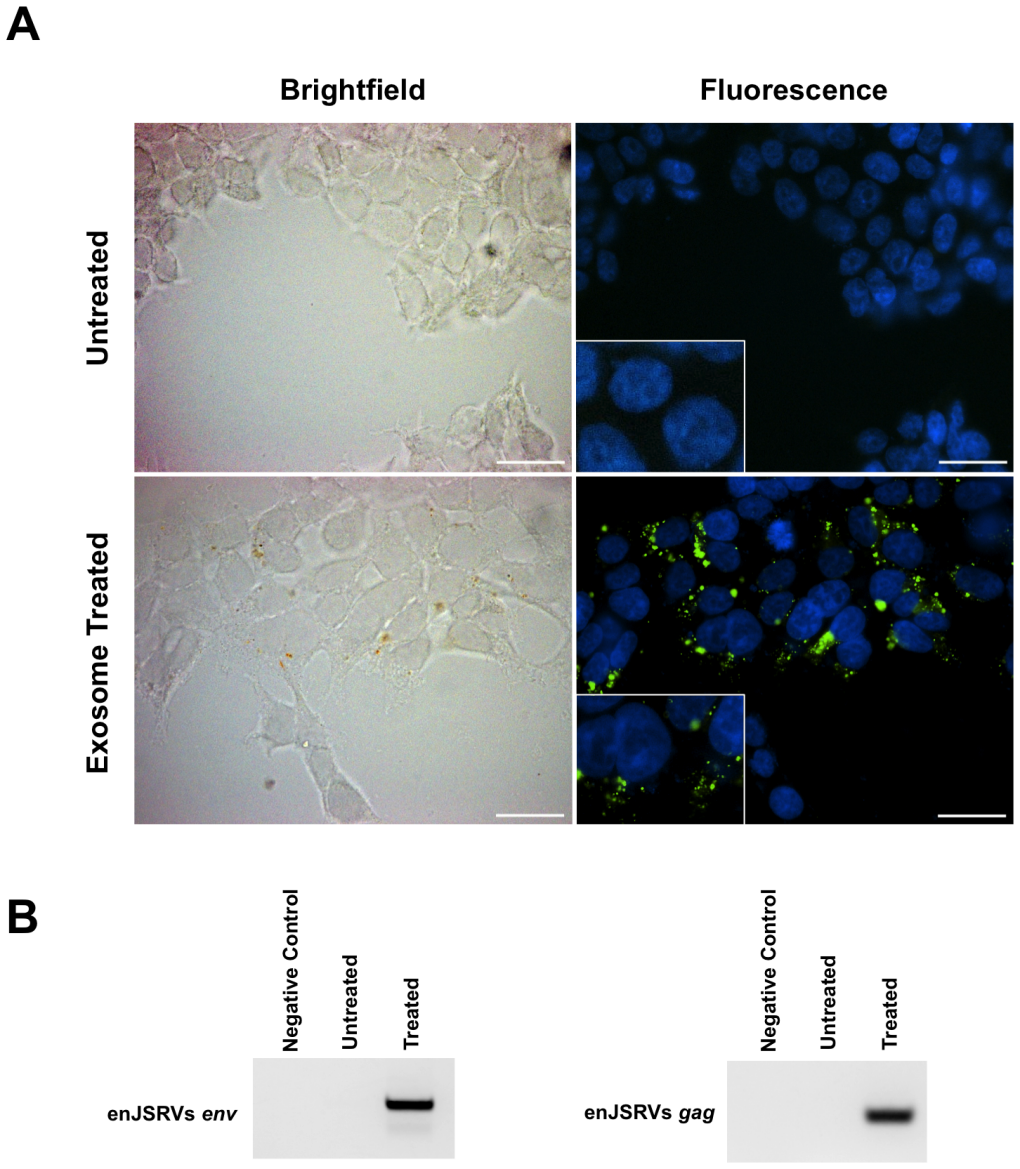
Delivery of enJSRVs *env* and *gag* RNA to HEK-293 cells. (A) Brightfield and epifluorescent images of HEK-293 cells, counterstained with DAPI, untreated or exposed to extracellular vesicles labeled with PKH67 green fluorescent linker dye. (B) RT-PCR analysis of cell lysate for enJSRVs *env* and *gag* 48 hours after treatment with labeled extracellular vesicles.

Both enJSRVs *env* and *gag* RNA were present in extracellular vesicles isolated from ULF of cyclic and pregnant ewes by RT-PCR analysis (Fig. 4B). In order to determine if the ULF extracellular vesicles could deliver these RNAs to cells, extracellular vesicles from pregnant ULF were isolated and labeled with a lipophilic fluorescent dye. The labeled extracellular vesicles were incubated with HEK-293 cells, which are of human origin and do not contain or express enJSRVs genes. As shown in Figure 7A, binding of the fluorescently labeled extracellular vesicles could be detected 72 hours post-incubation within HEK-293 cells. Additionally, orthogonal sections (Fig. S1) and a 3D rendering of z-stack images (Fig. S2 and Video S1) provide evidence of exosome uptake by HEK-293 cells. Further, RT-PCR analysis found that enJSRVs *env* and *gag* RNA in HEK-293 cells incubated with extracellular vesicles (Fig. 7B).

## Discussion

Results of these studies support the hypothesis that extracellular vesicles comprising both exosomes and microvesicles are a component of the uterine environment of cyclic and pregnant ewes. Electron microscopy, nanoparticle tracking analysis, and the presence of exosomal protein markers CD63 and HSP70 in the isolated ULF extracellular vesicles support the conclusion that exosomes and microvesicles are likewise present in ULF of day 14 cyclic and pregnant ewes. The tetraspanins CD9, CD63, and CD81 along with heat shock proteins HSP70 and HSP90 are typical exosomal marker proteins [38]. In addition to TEM, nanoparticle tracking analysis was used to quantify and determine the size of particles within each ExoQuick-TC isolated ULF sample. The size of the particles is consistent with the ExoQuick-TC precipitation procedure pulling down both exosomes (typically 30 to 100 nm in diameter) and microvesicles (>100 nm). Given that exosomes are typically 30–100 nm in diameter, it is likely that that the ExoQuick-TC reagent isolated larger microvesicles as well as exosomes. Indeed, the tail from these peaks extended to 300 nm, suggesting larger microvesicles were also present but in smaller amounts. However, both exosomes and microvesicles contain and deliver genetic material in the form of mRNA and miRNA to recipient cells [39].

Mass spectrometry analysis of the isolated extracellular vesicles found many proteins expressed by the endometrial epithelia and/or conceptus trophectoderm including cathepsin L1 (CTSL1), gastrin releasing peptide (GRP), lipoprotein lipase (LPL), myxovirus one (MX1), and prostaglandin synthase two (PTGS2) [12], [40]–[46]. The myxovirus resistance proteins MX1 and MX2 are expressed in the endometrium of both sheep and cattle in response to conceptus-produced IFNT, but their functions are currently unknown [44]. Indeed, an ovine endometrial glandular epithelial cell line produces and secretes extracellular vesicles *in vitro* containing the protein MX1 in response to IFNT [12]. Both CTSL1 and PTGS2 are expressed in the endometrial LE and superficial GE, whereas GRP is expressed in the LE and GE [40]–[42], [45], [46]. Extracellular vesicles in the ULF of cyclic and pregnant ewes likely emanate from the endometrial LE and GE. However, PTGS2 and CTSL1 are also expressed in the trophectoderm of the elongating conceptus. Thus, the extracellular vesicles from the ULF of pregnant ewes are likely produced by the trophectoderm and endometrial epithelia as the molecular content of exosomes includes proteins, mRNA and miRNA that are dependent on the cell type from which they originate [14], [47]. Many of the proteins found in the exosomes (e.g., CTSL1, PTGS2, GRP) have previously been found to be present in ovine ULF based on Western blot analysis [48]–[50]. Interestingly, many of those proteins, CTSL1 for example, do not have a signal peptide for classical secretion or are considered intracellular proteins. Thus, extracellular vesicles and microvesicles provide a unique mechanism for transmission of RNA and proteins between the conceptus and endometrium that is independent of classical secretion mechanisms and protects the contents from proteases in the ULF. This mode of transmission also would allow for protein transmission to the conceptus or epithelium directly without needing its receptor.

The RNA profiles from collected extracellular vesicles revealed the presence of a large number of small RNA species as compared to conceptus or endometrium samples. Significantly, human synctiotrophoblast cells produce extracellular vesicles containing miRNAs that are subsequently found in maternal circulations where they could alter maternal gene expression [51]. MicroRNAs have also been shown to be associated with preparation of the endometrium for implantation [52]. Additionally, knockdown of *Dicer* to reduce miRNA expression in placental explants enhanced cytotrophoblast cell proliferation suggesting a critical role in placental development for miRNAs [53]. Loss of *Dicer* is early embryonic lethal in mice with small and morphologically abnormal embryos collected at E7.5 [54]. Small RNA sequencing and comparison to the *Bos taurus* miRBase for mature miRNAs found 81 mature miRNA species and miR-423-3p unique to extracellular vesicles from day 14 pregnant ewes. Notably, miRNAs miR-7e, miR-7f and miR-451, were found to overlap with a recent study that investigated extracellular vesicles in the human uterine environment produced by an endometrial epithelial cell line [11]. A single miRNA can target many genes by eliciting degradation of mRNA or inhibiting mRNA translation [55]–[59]. Understanding the potential functional mechanisms of a miRNA is most efficient by classifying the targets. *In silico* analysis of miRNA-423-3p targets in *Bos taurus* showed target genes from a variety of biological processes including metabolism, immune system, cell cycle and apoptosis. A recent study utilizing human miR-423 found that hsa-miR-423-3p was capable of inducing cell proliferation without increased apoptosis, providing a possible role in increasing trophectoderm outgrowth during this period of rapid elongation of the conceptus [60]. In order to predict targets of biological significance, the miRNA expression profiles of the conceptus and endometrium must be combined with the transcriptome. Predicted targets can then be validated by *in vitro* binding studies or application of miRNA mimics and inhibitors. Future studies should therefore focus on the ovine endometrial and conceptus transcriptome during the periimplation period to determine miRNA targets.

We recently reported that enJSRVs are transmitted from the sheep uterus to the conceptus trophectoderm using a trans-species embryo transfer model [32]. In that study, the transmission of enJSRVs was hypothesized to occur via the endometrium shedding enJSRV particles that entered the conceptus via cell surface receptors, hyaluronidase two (HYAL2). The presence of enJSRVs *Env* and *Gag* RNA in exosomal contents provides a mechanism from the transfer from the endometrial epithelia to the conceptus trophectoderm prior to implantation as observed using a transspecies embryo transfer model [32]. In humans, recent work has also demonstrated the presence of *syncytin-1*, the envelope gene of an endogenous human retrovirus (HERV-W) involved in the fusion of trophoblast cells, in extracellular vesicles collected from placental explants and primary trophoblast cultures [19], [61]. The presence of ERVs in extracellular vesicles from both humans and sheep suggests a conserved mechanism of pre-implantation signaling from the endometrium to the developing conceptus trophectoderm.

The results of this study support the idea that exosomes and microvesicles are present in ULF of pregnant and cyclic ewes, contain specific proteins, miRNAs, and mRNAs, and are capable of delivering their contents *in vitro*. Additionally, the molecular contents varied by pregnancy status suggesting differential loading of extracellular vesicles emanating from the endometrial epithelia or the distinct presence of extracellular vesicles derived from the conceptus trophectoderm; either instance serving as evidence of pre-implantation communication of the conceptus and endometrium via cell secreted or shed vesicles. Discovery of enJSRVs RNAs in extracellular vesicles provides an important mechanism for the delivery of retroviral transcripts to the trophectoderm that are critical for conceptus development prior to implantation. Importantly, these studies support the idea that extracellular vesicles in the ULF have a biological role in conceptus-endometrial interactions important for the establishment and maintenance of pregnancy.

## Supporting Information

Table S1 **Result tables from Nano LC-MS/MS proteomic analysis of cyclic and pregnant ULF extracellular vesicles.**
(XLSX)

Figure S1 **Orthogonal sections of HEK-293 cells treated with labeled ULF extracellular vesicles.** Epifluorescent z-stack images of HEK-293 cells, counterstained with DAPI, and treated with extracellular vesicles labeled with PKH67 green fluorescent linker dye. Orthogonal sections demonstrate the presence labeled vesicles throughout the cell monolayer. Scale bar = 20 µm
(TIF)

Figure S2 **3D image of HEK-293 cells treated with labeled extracellular vesicles.** Scale bar = 20 µm
(TIF)

Video S1 **Rotating 3D image of HEK-293 cells treated with labeled ULF extracellular vesicles.**
(MP4)
