# Supplementary figures and images for: Extracellular Vesicles in Luminal Fluid of the Ovine Uterus
Source: PLoS One. 2014 Mar 10;9(3):e90913. doi: 10.1371/journal.pone.0090913 (PMC3948691; doi:10.1371/journal.pone.0090913)

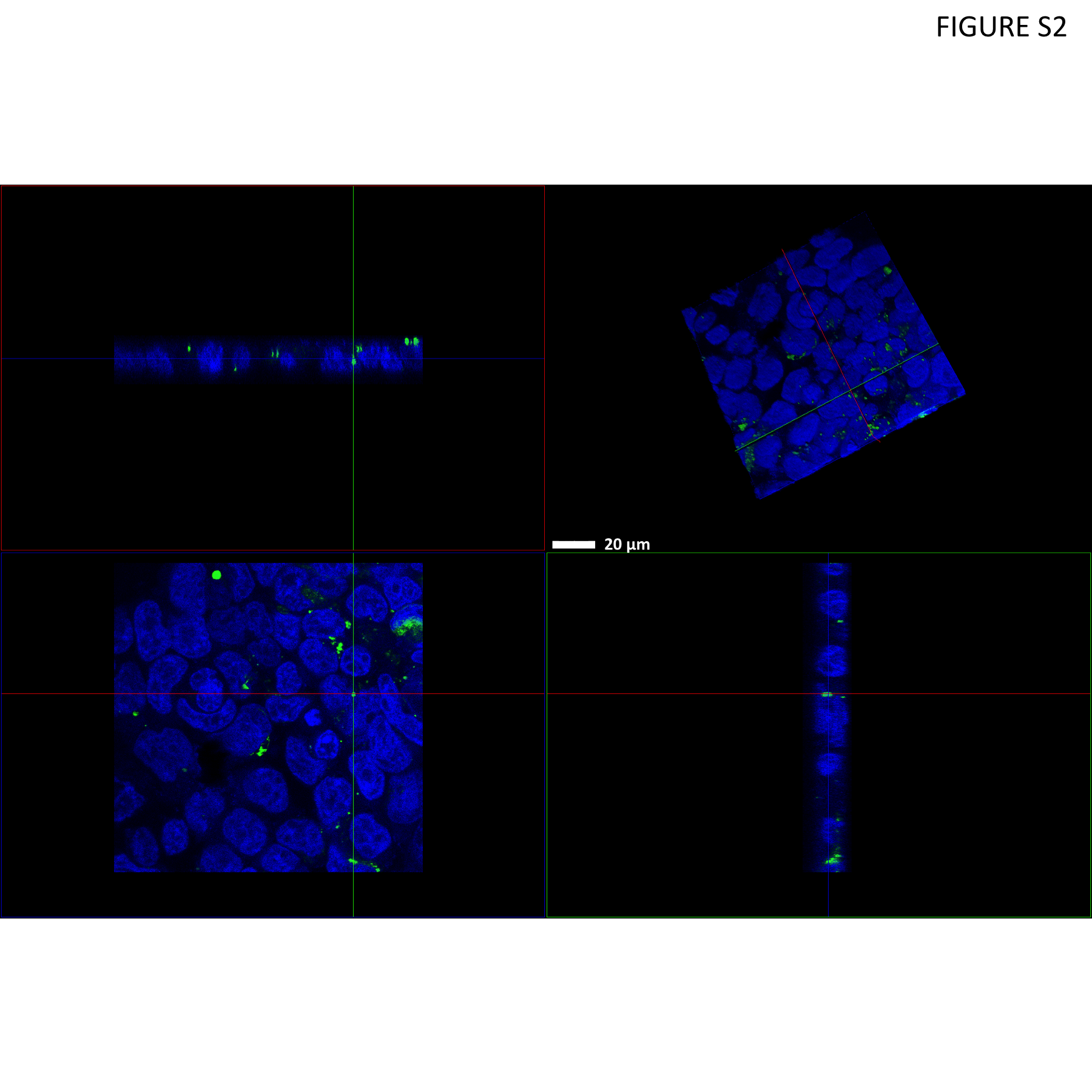

Supplement: Figure S1 — Orthogonal sections of HEK-293 cells treated with labeled ULF extracellular vesicles. Epifluorescent z-stack images of HEK-293 cells, counterstained with DAPI, and treated with extracellular vesicles labeled with PKH67 green fluorescent linker dye. Orthogonal sections demonstrate the presence labeled vesicles throughout the cell monolayer. Scale bar = 20 µm (TIF) [file pone.0090913.s002.tif]

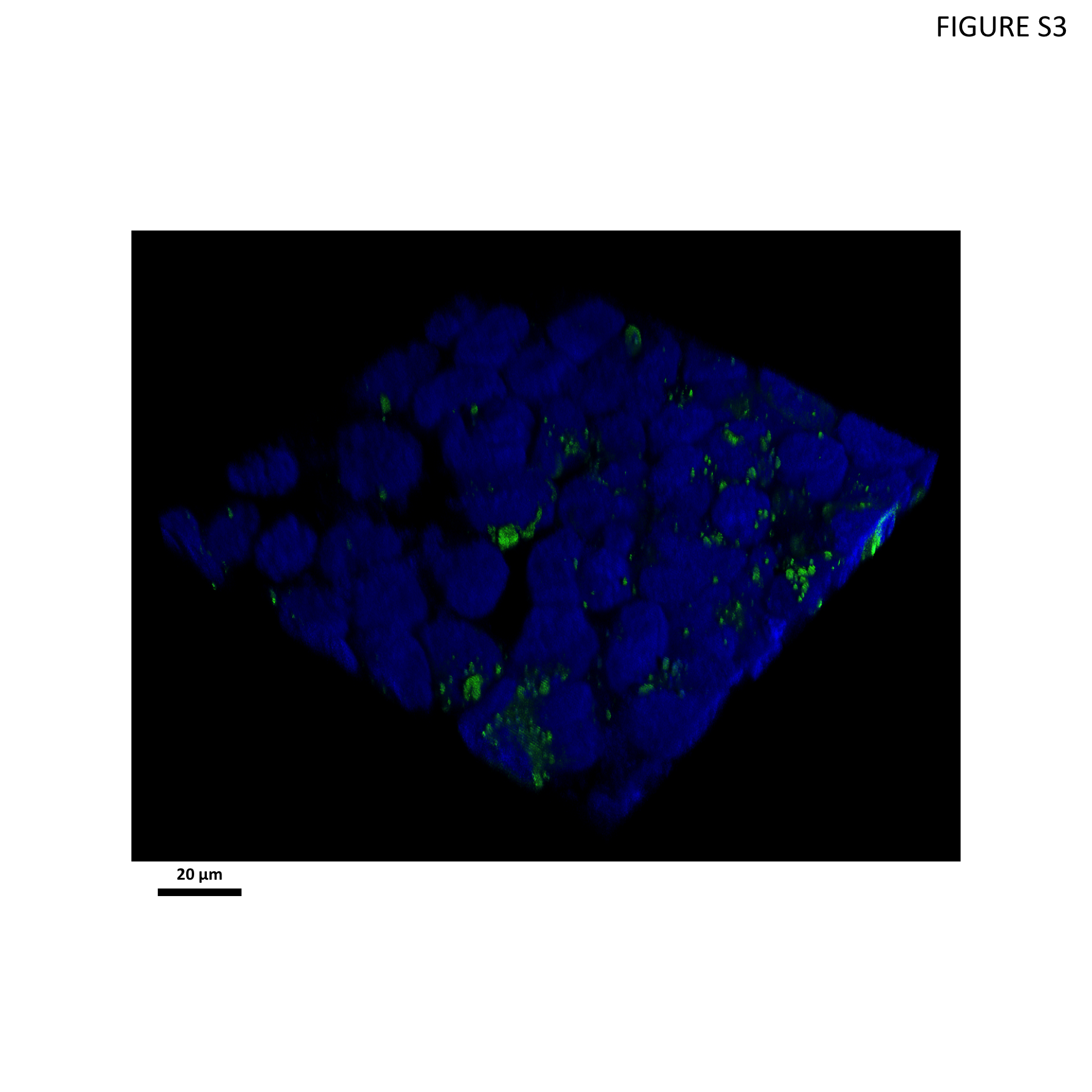

Supplement: Figure S2 — 3D image of HEK-293 cells treated with labeled extracellular vesicles. Scale bar = 20 µm (TIF) [file pone.0090913.s003.tif]
